# Prescriptions and Dispensing

**Published:** 1907-12-21

**Authors:** 


### Therapeutics.

### PRESCRIPTIONS AND DISPENSING.

Incompatibility (*continued*).

It by no means follows, of course, that in every case, such as the above, in which two soluble ingredients lead to the formation of an insoluble compound which is precipitated, that the prescription is erroneous. The following is an example to the contrary:—

| | | |
|---|---|---|
| ℞ Tincturæ Ferri Perchloridi | ... ... | ♏lxxx. |
| Syrupi | ... ... ... ... ... | ℥ss. |
| Spiritus Ammoniæ Aromatici | ... ... | ʒij |
| Aquam | ... ... ... ... | ad ℥viij. |

Reaction occurs between the alkali and the iron, and ferric hydroxide is precipitated. If it is the intention of the doctor to give freshly prepared ferric hydroxide, the prescription is a good one; if, on the other hand, perchloride of iron itself was intended, for styptic purposes, for example, then the prescription is a bad one. It remains only to add that in the former case the reacting ingredients should be diluted separately before they are mixed, to ensure fine subdivision of the precipitate.

Although the question of how to deal with incompatible ingredients may arise in connection with other forms of medicine besides the liquid, it is of much more frequent occurrence in the cases of mixtures, lotions, and liniments than in others, and especially in that of mixtures. The reasons for this are not difficult to discover; the very great variety of drugs that are ordered in this form more or less frequently, and the favourable conditions for chemical reaction offered by their presence in a liquid medium, are enough to account for it. Before we pass on to the special group of mixtures known as emulsions, it may be well to discuss a few typical cases of incompatibility.

*Reaction Between Salts.*

As students of analytical chemistry are well aware, the production of a precipitate on mixing solutions of two salts is an extremely common occurrence. When this occurs in dispensing, it is usually a simple matter to decide what is happening, and whether it ought to be prevented or not. We have just given an example in which it is intentional, and in our last article we gave another in which a strychnine salt was one of those reacting, and in which the precipitate was not merely undesirable, but also actually dangerous. We will give a few other cases:—

| | | |
|---|---|---|
| ℞ Zinci Sulphatis | ... ... ... ... | gr. xl. |
| Liquoris Plumbi Subacetatis Fortis | ... | ℥iss. |
| Tincturæ Opii | ... ... ... ... | ℥ss. |
| Aquam | ... ... ... ... | ad ℥vi. |

In this lotion the prescriber has evidently overlooked the reaction that will occur between the lead subacetate and the zinc sulphate, all the lead being precipitated as insoluble sulphate and thus rendered useless. The same remarks apply equally to the next example, but there it is the zinc that is precipitated, as borate:—

| | | |
|---|---|---|
| ℞ Sodii Biboratis | ... ... ... ... | gr. xx. |
| Zinci Sulphatis | ... ... ... ... | gr. v. |
| Aquam Rosæ | ... ... ... | ad ℥iij. |

A simple reaction between two ingredients may cause a good deal of trouble even when no actual precipitate results. The following is a fairly common case:—

| | | |
|---|---|---|
| ℞ Ammonii Carbonatis | ... ... ... | gr. xx. |
| Vini Ipecacuanhæ | ... ... ... | ʒiss. |
| Syrupi Tolutani | ... ... ... ... | ʒiij. |
| Syrupi Scillæ | ... ... ... ... | ʒiij. |
| Infusum Senegæ | ... ... | ad ℥iv. |

Syrup of squills contains free acetic acid, which acts on the ammonium carbonate with evolution of carbon dioxide; this is produced very slowly, and the syrupy constituents of the mixture cause much froth to be formed. The syrup of squills, if used at all, should be diluted with part of the infusion, or, if concentrated infusion is to be used, with water, and added to the powdered ammonium carbonate in the bottle; the latter may then be gently warmed, set aside lightly corked, and gently shaken at intervals until the reaction is complete. It would be much simpler, of course, not to use syrup of squills at all, but equivalent quantities of simple syrup and tincture of squills, leaving out the acetic acid altogether unless it were very particularly desired.

*Precipitation of Alkaloids.*

Mercuric chloride and potassium iodide are often prescribed together in solution, and the resulting mixture is virtually dilute Mayer's solution; it is, therefore, a precipitant of alkaloids, and yet the latter are not infrequently ordered in the same mixture, as in the following instance:—

| | | |
|---|---|---|
| ℞ Liquoris Hydrargyri Perchloridi | ... | ℥iss. |
| Potassii Iodidi | ... ... ... ... | ʒij. |
| Syrupi Zingiberis | ... ... ... ... | ℥ss. |
| Infusi Cinchonæ Acidi | ... ... ... | ℥ij. |
| Aquam | ... ... ... ... | ad ℥viij. |

The alkaloids of the cinchona are precipitated as by Mayer's solution; the precipitate contains much of the mercury, and it is important that the patient shall not get too much in one dose. To prevent this as far as possible, if the prescription is maintained, the syrup of ginger and half the water should be added to the decoction of cinchona; the other half of the water to the other ingredients, and the liquids mixed with only gentle shaking.

It must be remembered that most alkaloids are precipitated not only by alkaline hydroxides and carbonates, but also by salts which are alkaline in nature though not in constitution, such as ordinary sodium phosphate; in such cases, if there is no particular object in keeping the medicine alkaline, the alkaloids may be re-dissolved by the addition of just sufficient acid for the purpose.

*Precipitation by the Vehicle.*

Precipitation may occur, not by the production of a new substance by reaction, but from the composition of the vehicle. One of the commonest cases of this is when a salt, soluble in water, is precipitated by the presence of a quantity of tincture or other alcoholic liquid; there is then a good deal of probability that the precipitate will be in crystalline form, or even in large crystals. It is necessary to foresee such an occurrence, and accelerate it by dissolving the salt in the least possible quantity of water and adding this solution to the alcoholic liquids, in order that the precipitate may be reduced to the finest possible powder, and the bottle labelled "Shake." The following presents a somewhat similar case:—

| | | |
|---|---|---|
| ℞ | Ferri Phosphatis (scale) ... ... ... | ʒij. |
| | Syrupi Zingiberis ... ... ... ... | ℥ss. |
| | Acidi Phosphorici diluti ... ... ... | ℥ss. |
| | Aquam ... ... ... ... ad | ℥viij. |

The scale phosphate of iron, which is not officinal, contains ferric phosphate and sodium citrate, and it is soluble in water; but on the addition of dilute phosphoric acid a precipitate of ferric phosphate is produced. This is soluble in a large excess of acid, but the amount of the latter required is more than could justifiably be given to the patient.

In the next prescription a similar result is produced by double decomposition:—

| | | |
|---|---|---|
| ℞ | Ferri et Quininæ Citratis ... ... ... | ℈j. |
| | Acidi Phosphorici diluti ... ... ... | ℈j. |
| | Tincturæ Cardamomorum compositæ ... | ʒiij. |
| | Syrupi Limonis ... ... ... ... | ʒij. |
| | Aquam ... ... ... ... ad | ℥ij. |

In this case the precipitation may be almost, or quite, prevented by dissolving the scale salt in two-thirds of the water with the syrup of lemon, and diluting the phosphoric acid with the rest of the water before mixing. The tincture should be added last.

